# Influence of HRM on CSR and performance of upscale hotels in developed and developing countries

**DOI:** 10.1007/s10668-022-02711-x

**Published:** 2022-10-17

**Authors:** Huma Sarwar, Muhammad Ishtiaq Ishaq, Simona Franzoni

**Affiliations:** 1 Department of Economics and Management, University of Brescia, Brescia, 25122 Italy; 2 Quaid-i-Azam School of Management Sciences, Quaid-I-Azam University Islamabad, Islamabad, 45320 Pakistan

**Keywords:** Corporate social responsibility, Human resource management, Sustainable performance, Upscales hotels, Cross-cultural study

## Abstract

The researchers showed their increased interest in linking human resource management (HRM) and corporate social responsibility (CSR) practices in recent studies. HRM is a critical factor in how CSR should be enacted, developed, and understood at a broader level to achieve organizational benefits. Hence, it is evident that current studies are asking for more studies on HRM–CSR nexus, and we argue that such a relationship is necessary and relevant. Probing more in this research stream, the current research investigates the impact of HRM and CSR on sustainable performance. More specifically, this study explores how 3-star, 4-star, and 5-star hotels achieve sustainable performance through HRM and CSR in the hospitality industry of the UK, Italy, and Pakistan. A stratified random sampling technique was used to select the hotels by collecting hotel details from Chambers of the Commerce United Kingdom, Italian Government Statistics, and Pakistan Hotel Associations for the UK, Italy, and Pakistan. Through a highly structured survey questionnaire, the data were collected from 438 UK, 520 Italian, and 354 Pakistani hotels. The results confirm the HRM–CSR–sustainable performance nexus in the hospitality industries of three countries. They show that HRM impact on CSR and sustainable performance is relatively stronger in five-star hotels followed by three-star and then four-star hotels. These results supported the resource-based view theory by providing strong evidence that HRM and CSR are essential resources for achieving sustainable performance and competitive advantage.

## Introduction

In the twenty-first century, the global economy is driven by three major industries: tourism, telecommunication, and technology (Khuong & Linh, 2020). It is easily claimed that the tourism industry is considered the world’s largest economic sector as it generates jobs and accounts for a significant proportion of the world’s GDP (Vasquez, 2014). According to the record of WTTC, in 2019, Travel and Tourism’s total contribution to the global economy was 10.3% that was a rise of 2.5% than 2018, and it is anticipated that this contribution will continue to grow in 2020. It is forecast to rise by 3.7% to USD 13,085.7 bn by 2029 (11.5% of GDP). In total, by the Travel & Tourism report to employment 330 million jobs in 2019 were recorded. By 2029, Travel & Tourism is forecast to support 420,659,000 jobs (11.7% of total jobs), increasing 2.5% pa over the period. But, the COVID-19 pandemic seriously halted this growth by suffering a 4.7 USD trillion loss in 2020 and a 3.7% GDP decline in the world’s economy. In alone 2020, a total of 18.5% of jobs were lost (i.e., roughly 62 million jobs) around the globe, whereas international visitors’ spending was reduced by 69%, whereas domestic spending decreased by 45% (WTTC, 2021).

As the hospitality industry is considered one of the fastest-growing industries around the globe and plays a critical role in promoting tourism, this industry provides new experiences, sharing of knowledge, job creation, leisure and business travel, and economic and social benefits for society. But this sector is also responsible for its adverse impact on the economic, social, and natural environments such as waste generation, biodiversity loss, noise pollution, air pollution, and climate change (Chung & Parker, 2010). Some other challenges are pricing elasticity, financing costs, eco-friendly practices, licensing issues, and taxes (Ishaq et al., 2022). Therefore, most of the hotels started facing pressures from different stakeholders such as consumers, pressure groups, to pay attention to social responsibility issues and environmental concerns by providing eco-friendly service that is not harmful to the natural environment and is safer for the stakeholders, primarily the corporate social responsibility (CSR) related initiatives. Apart from environmental issues, the stakeholders also ask the hospitality industry to play a critical role in clean energy, well-being, global warming, poverty reduction, responsible production, and consumption. Therefore, this industry is now forced to review its strategies for addressing environmental and social issues to achieve sustainable performance.

Recent years have seen an increasing interest in the concept of CSR in the management literature (Pino et al., 2016; Zeng, 2020; Zhu & Zhang, 2015). CSR is considered the organizational commitment to sustainable environmental, social, and economic development to improve the quality of life of people, including workforces, their families, and the local community (Dey et al., 2018; Garcia-Sanchez & Araujo-Bernardo, 2020). Furthermore, CSR is seen as an organizational activity associated with business ethics, meaning the obligation of the corporational operations to environmental and social concerns (Wang et al., 2015). These sustainable practices force organizations to pay attention to CSR initiatives to achieve sustainable growth objectives (Gorski et al., 2017). Overall, CSR has become an increasingly important subject in all industries, including the hospitality industry. In practice, many hotels are also trying to play their role in less harmful practices for the society and environment (Dahlsrud, 2008; Liu et al., 2014; McWilliams et al., 2006).

The implementation of CSR activities is related to the considerable changes in the firms (Dunphy et al., 2007). It involves adapting existing practices, policies, and strategies to align CSR values with employees’ behavior and the routine business agenda (Wheeler et al., 2003; Zadek, 2007; Dubois & Dubois, 2012; Nazir & Ul Islam, 2020; Bibi et al., 2021). Therefore, the effectiveness of CSR practices depends on the way the firms adopt them. It requires support from the organization’s management, as human resource management (HRM) has long been concerned with deciding how CSR activities should be identified, developed, and implemented (Srinivasan & Arora, 2015). HRM plays its role in developing practices, approaches, policies, and strategies to assist organizations in handling social, economic, and environmental challenges (Leroy et al., 2018). The researchers argued that the HRM enables businesses to strengthen them in a rapidly changing environment and support their legitimacy by playing a vital business partner role (Brockbank & Ulrich, 2009; De Gama et al., 2012; Leroy et al., 2018).

With the newfound and intensifying interest and relevance in CSR activities and HRM strategies in the tourism and hospitality industry, there is a dearth of research on these practices and their impact on sustainable performance. The nexus of HRM–CSR has been extended (Jamali et al., 2015), and HRM's role in enabling and contributing to the persuasive transformation toward socially responsible and more sustainable organizations remains unclear. The extent to which HRM's actions could impact developing and guiding CSR initiatives and achieving sustainable performance is an important question to be addressed. Moreover, the economic differences also played an essential role in formulating and implementing strategies. Considering these research gaps, this study examines the relationship of HRM and CSR in achieving sustainable performance in 3-star, 4-star, and 5-star hotels of the UK, Italy, and Pakistan. More precisely, this research addresses the following research questions:

- **RQ1**: Does HRM influence CSR in hospitality industry?
- **RQ2**: Does CSR play a role in achieving sustainable performance?
- **RQ3**: Do HRM’s impacts on CSR and sustainable performance differ in 3-star, 4-star, and 5-star hotels?
- **RQ4**: Does the relationships among HRM, CSR, and sustainable performance differ in the UK, Italian, and Pakistani hospitality industry?

The structure of the paper is as follows. In the following sections, the supporting theory and literature review section was explained. Section 3 addresses the research methodology, including sample and sampling techniques, data collection method, and measurement instruments. The result of this study is discussed in Sect. 4, whereas discussion, recommendations, policy implications, and the conclusion are discussed in Sect. 5.

## Literature review

### Resource-based view theory

CSR concept can be conceptualized based on multiple aspects such as social performance (Carroll, 1979, 2020), business ethics (Solomon, 1993), corporate governance (Freeman & Evan, 1990), and accountability (Elkington, 1998). The researchers used different theoretical frameworks to address the CSR issues, such as Stewardship theory, Stakeholder Theory, Agency Theory, Institutional Theory, and Social Identity Theory. Among these theories, Branco and Rodrigues (2006) were among the first authors who used resource-based view (RBV) theory to address CSR. They argue that RBV can address CSR more comprehensively because of the significance of intangible resources and capabilities to achieve competitive advantage. Wernerfelt introduced the RBV theory in 1984, and Barney subsequently developed that in 1991. They argue that the resources and capabilities that are rare, non-substitutable, valuable, and inimitable are considered a source of competitive advantage. The resources include human capital (employees’ intelligence, judgment, and skills), organizational capital resources (HR systems, control systems, and organizational structure), and physical resources (finance, equipment, and plants) (Barney, 1991). Among others, intangible resources include reputation, human capital, technology, organizational culture, and HRM (Grant, 1991; Surroca et al., 2010).

The applicability of RBV theory in this research is due to the extent of literature supporting the notation of competitive advantage using both HRM and CSR. For instance, in an assessment of 166 studies on RBV, only two percent of studies partially support the RBV stance. In contrast, the rest of the studies strongly support the logic of RBV in addressing various factors such as knowledge, environmental performance, financial performance innovation, and human resources. Secondly, the strategic role of HRM accentuates the significance of HRM in any organization that leads to enhanced organizational performance and acts as a catalyst for sustained competitive advantage (Barney, 2001). In a recent competitive environment, human resources are concepted as an inextricable and indispensable part of an organization that leads to profitable investments and competitive advantage.

### Human resource management

The scholars have constantly asked for new studies on both micro-level and macro-level antecedents of CSR (McWilliams et al., 2006; Rodriguez et al., 2006; Rupp et al., 2006). The collective efforts within the organization to create and support CSR activities with ownership and motivation are known as internal support to CSR. Such initiatives cannot succeed or prevail within the organizations if employees do not support such policies with motivation. González-Benito and González-Benito (2010) asserted that organizations must disseminate proper and up-to-date information to all internal stakeholders to create and implement CSR strategies effectively. Lee et al. (2018) identified that the employees are related to the organization’s CSR activities and attached themselves to the firm. The researchers (Aguilera et al., 2007; Logsdon & Yuthas, 1997; Maignan & Ralston, 2002) claimed that organizations’ moral development, employee power, network centrality, and employees’ beliefs and top management enthusiasm are theoretically. In an empirical study, De Luque et al. (2008) find that top-management importance on stakeholder or economic values is observed by employees and creates a sense of whether their organization is more visionary or autocratic. The employees may foster a positive perception about the organization if managers (the role of HRM) mentioned CSR practices in their communications, integrated them into objectives, and encouraged their teams to incorporate them (Bhattacharya et al., 2008; Lichtenstein et al., 2004).

HRM is considered the organizational activities associated with developing, managing, and maintaining human resources (Jamali et al., 2015). It mainly involves organizations' options from the given plans, techniques, and frameworks for handling staff members. While there is no clear-cut meaning of HRM, it is usually referred to as the intended exercise of tasks for personnel meant to allow an organization to accomplish its objectives by monitoring the individuals and their job performance (Boxall et al., 2007). Ehnert et al, (2016, p. 90) defined HRM as “the adoption of HRM strategies and practices that enable the achievement of financial, social and ecological goals, with an impact inside and outside of the organization and over a long-term time horizon.” Boxall, (2007), also defined HRM as the relationship between employees and employers contribute to the organizational goals efficiently and effectively over the long term. Thus, HRM is explained as a set of people-oriented functions in an organization developed purposefully to increase employee effectiveness. According to Purcell (2014), HRM is constructed into the “wiring of organizations” because all organizations that utilize work count on some human resourcing procedure and include the inevitable job of taking care of their employees and the job they do. It is concentrated at both collective and individual levels and consists of initiatives to take care of employees and create an effective working environment (Voegtlin & Greenwood, 2016). Senyucel, (2009, p. 25) defined HRM as a “combination of people-cantered management practices that recognizes employees as assets and those that are geared to creating and maintaining a skillful and committed workforce for achieving organizational goals.”

Boxall et al. (2007) also proposed different techniques to measure HRM strategies and practices, including society, business unit, industry, organization, hierarchy, and occupation that differ according to the wide range of organizational activities, behaviors, strategies, and policies. It is believed that human resource practices/strategies are mainly linked within an organizational operation. Still, the literature proposed that such strategies are more effective if seen in a broader context. Purcell (2014) and Guest and Bos-Nehles (2013) also support the above notation that HRM activities are not developed or controlled within an organization. Still, they are applicable in both internal and external operations such as competitive strategies, organizational size and structure, social and economic environment, trade unions, corporate cultures, and legislations.

### Corporate social responsibility

CSR is how organizations incorporate economic, environmental, and social concerns into their culture, values, decision-making, operations, and strategy in an accountable and transparent manner, consequently setting up better practices, making money, and improving society (Zeng, 2020). The CSR-related programs are focused on environmental, social, and economic influence on organizational activities that are performed beyond the profit maximization context (Chowdhury et al., 2019; García-Sánchez & Araújo-Bernardo, 2020). The researchers (e.g., Armstrong, 2008; Dey et al., 2018) claimed that CSR is a commitment and mission of an organization to run its businesses ethically and support the society and environment in which the company is operational. CSR is also defined as the voluntary integration of social and environmental concerns into an organization’s practices, strategies, and values (Cruz & Pedrozo, 2009).

The World Business Council sets a comprehensively cited definition of CSR for Sustainable Development, which indicates that CSR is the lasting commitment by the corporation to perform activities ethically, important for the economic development in terms of improving the quality of life of workers, their families as well as local communities and society as a whole (Richard & Okoye, 2013). In line with this concept, it is proposed that CSR be effectively and efficiently implemented. It needs to involve employees and be incorporated into its culture, values, strategy, and objectives. Inyang et al. (2011) also argue that CSR initiatives and strategies come to life through decision-making and employee activities.

CSR must go beyond community projects and philanthropy and consider how businesses influence the society and environment in which it is operational (Jamali et al., 2017; Michailides & Lipsett, 2013; Sharma et al., 2015). It is believed that CSR should be incorporated in organizational practices as a strategy rather than considering it as a philanthropic activity only. We believe that employees have a significant role in implementing CSR effectively and efficiently and incorporate it in organizational mission, values, objectives, and culture. Lapiņa et al. (2014) claimed that socially responsible organization shows that the company abide by all the legal requirements and invest in human capital and environmental issues.

However, Garavan et al. (2010) articulated that developing and implementing CSR strategies depend on the discretionary behavior of employees. They also argued that difficulties to effective CSR result from perceived organizational support, employees' lack of understanding and knowledge and perspectives toward CSR. Lam and Khare (2010) suggest that legitimate and effective CSR initiatives and strategies need integration into an organization's culture, processes, structure, and strategy. Moreover, strategic CSR has a positive role in achieving competitive advantage and higher performance (Carroll & Shabana, 2010; Emmott & Worman, 2008).

### Sustainable performance

The concept of performance or sustainable performance has various contexts. For instance, Nizam et al. (2019) explained sustainable performance (financial, social, and environmental performance) as the utilization and management of a firm’s resources in a way that leads to maximum environmental, economic, and social benefits. They further explained that this effective and efficient consumption of a firm’s resources should simultaneously meet current demand's needs and enhance and protect future generations' opportunities. Sustainable performance is linked with financial performance, customer retention, and growth by reducing their negative influence on society and the environment. Chen et al. (2012) defined sustainable performance as the organizational practices that maximize performance without harming the environment (Latan et al., 2018).

The rapid development of economic expansion has had a devastating impact on the world’s natural resources and the whole environment. According to Elkington (1998), with the increasing focus on sustainable performance and social responsibility, companies have set themselves new objectives, not merely thinking about the economic benefits such as a commitment to environmental and social outcomes. Kiron et al. (2012) surveyed 2800 global companies showed that 70% of these companies consist of sustainability as a critical issue in their strategic policies and agendas. The United Nations General Assembly proposed the 2030 Agenda that contains 17 sustainable development goals (SDGs) that are further categorized into 169 different targets.

From the business viewpoint, the primary aim behind SDGs is to crate innovation, people-oriented and sustainable economies to increase employment opportunities. The organizational goal is to ensure that their employees are well educated and healthy and support the proficiencies, understanding, and effectiveness required to create a proactive citizen, productive workforce contributing to society. The achievement of SDGs requires a strategic process between different agents such as individuals, philanthropic organizations, governments, non-governmental organizations, multinational enterprises, and the public and private industries. Interaction and collaboration between these agents will contribute a step further toward building harmonious culture, environmentally friendly production, and sustainable consumption. The SDGs (2030) agenda states itself as “an Agenda of the people, by the people, and for the people, and this will ensure its success” (United Nations, General Assembly, 2015). Therefore, we recognize the dual role of employees as originators and recipients of SDGs creation and implementation.

### Hypotheses development

Over the past decade, the awareness of sustainable practices has increased. The emerging CSR–HRM literature toward the sustainable practices has addressed the relationship between CSR–HRM strategies and employee work behaviors (Shen & Benson, 2016), indicating that HRM strategies and CSR activities together can contribute to organizational performance in the following areas: task performance, organizational identification organizational commitment hiring of quality employees, employee retention, employee morale, organizational identification, employee engagement through volunteerism programs, and countering the possibility of adverse employee behavior (Bhattacharya et al., 2008; Boğan, et al., 2018; Farooq et al., 2014; Meier et al., 2021; Shen & Benson, 2016).

The researchers argued that HRM played its critical role in highlighting the importance of CSR to top management and convinced them to design such strategies in light of organizational competencies and capabilities, execute them effectively, and also promote such culture among their employees (Jamali et al., 2015; Parkes & Davis, 2013; Voegtlin & Greenwood, 2016). Therefore, the firms continuously adopt numerous strategies to retain the best talents to develop a competitive advantage in the business environment. Progressively, the firm’s management understands the significance of CSR to attract and hold the best employees that may lead to better organizational performance (Goyal & Kumar, 2017). As stated by Kundu and Gahlawat (2015), HR strategies and practices may help firms motivate the workforce to perform more effectively, resulting in improving organizational performance.

Extant literature proposed HRM presents the greatest potential for the incorporating the CSR initiatives and activities as compared to other factors such as top management support (Jamali et al., 2015; Sharma et al., 2015). Increasing interest in sustainable activities in the organizations is related to the increasing pressure from customers, communities, governments, and non-governmental authorities, to develop more accountable toward society and environment and to be more actively involved in the solution of social and environmental concerns (Franzoni et al., 2021). Many other authors have stated that HRM can engage with CSR from different perspectives, for instance, training development, motivation, recruitment of employees and employee well-being. Barrena‐Martínez et al. (2017) conducted comprehensive research on the Spanish context and found eight HRM practices with a social orientation: such as work condition, equal and diversity opportunities, training, social protection, social dialogue, management of labor relations, equity in remuneration, occupational health, and safety, working conditions and social protection. Similarly, research developed by Bučiūnienė and Kazlauskaitė (2012) observed five HR policies with an alignment concerning CSR activities: health and well‐being, flexible working hours, involvement of minority employees, ownership shares and communication and job rotation.

Jamali et al. (2015) argued that deeper integration of the two domains provides synergies, resulting a shared creation of value for the many stakeholders. Similarly, Aguilera et al. (2007) explained in their research that the role of HRM in CSR can have a high significance and positive impact on the individual level of CSR. The incorporation of CSR concepts and initiatives into HRM practices and systems react to workers' CSR expectations and thus contribute to the development of positive employee attitude and behaviors, which in turn affect organizationally relevant outcomes such as employee job performance, satisfaction, and sustainable performance. Voegtlin and Greenwood (2016) and Orlitzky et al. (2006) argued that organizations should focus on CSR practices for better sustainable performance. Overall, it is suggested that HR impacts the organizational processes, positioning CSR initiatives and increasing organizational performance (Inyang et al., 2011). Based on the literature, the following hypotheses are proposed:

- **H1**. HRM plays a positive role in shaping CSR initiatives.
- **H2**. HRM has a significant role in achieving sustainable performance.
- **H3**. CSR positively influences sustainable performance.
- **H4**. CSR mediates the relationship between HRM and sustainable performance.
- **H5**. The impact of HRM on CSR is relatively different concerning hotel categorizations—3-stars, 4-stars, and 5-stars.
- **H6**. The impact of HRM on sustainable performance is relatively different concerning hotel categorizations—3-stars, 4-stars, and 5-stars.
- **H7**. The impact of CSR on sustainable performance is relatively different concerning hotel categorizations—3-stars, 4-stars, and 5-stars.

**Fig. 1:**
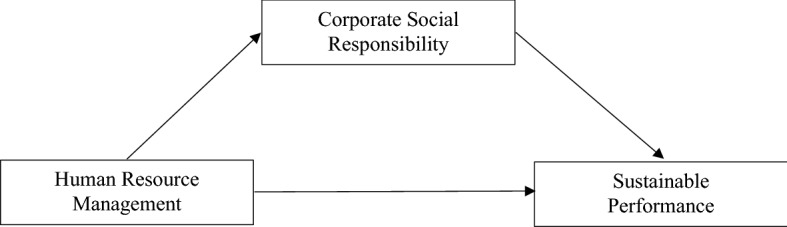
Conceptual framework

The significant contribution of the current paper provides new understandings and research synthesis in the academic literature on the relationship of HRM with CSR and performance. The data were collected from the hospitality industry in three countries. The results confirm that the impact of HRM on CSR and sustainable performance differs significantly between hotel categories. Using resource difference and availability of strategic resources within different hotel categorizations, this study tests a conceptual model (presented in Fig. 1) in a unique context that provides empirical support on how resources play its role in achieving sustainable performance in the hospitality industry.

## Methods and material 

### Study context

This research is cross-cultural in context in which we aim to highlight the difference between HRM, CSR, and sustainable performance nexus in hospitality industry. We have collected the data from UK, Italy, and Pakistan. Although we do not seek to introduce any cultural aspect to this research, the cultural and economic differences between developing and developed world explain different thoughts to the nexus of HRM, CSR, and sustainable performance. Mostly, US-based theories had implemented in developing and emerging economies, due to which the theoretical contribution and managerial implications are minimal (Ishaq et al., 2022; Sarwar et al., 2020).

Moreover, there are significant cultural differences between the countries under investigated. Using Hofstede (1991) framework, UK, Italy, and Pakistan have different ratings, especially on individualism and collectivism dimension. The people from collectivistic societies prefer to remain in groups while the people from individualistic societies are more interested in taking care of themselves. On this dimension, the UK scored 89 and consider the highly individualistic society after the USA and Australia. The UK system governs in a way that every individual is supposed to achieve personal goals and attainment of happiness through personal fulfillment. On the other hand, Pakistan score 14 and considered as one of the highly collectivistic society. The people in Pakistan manifest in extended relationships, extended family, be with family, and long-term commitments with the group members and societies. The collectivistic society considers loyalty as a paramount factor and overshadows regulations and societal rules. Such societies strive for a strong relationship in which every member takes responsibility for other group members. In the case of Italy, the society is considered an individualistic society with a typical “me” approach as it scored 76 on Hofstede’s cultural framework. Hofstede (1991) explained that Italians consider their life happiness via personal fulfillment and motivation through personal goals and objectives.

Considering these significant economic and cultural differences, this study addresses the one of the critical issues of cultural differences on individuals, groups, and organizations. Another difference along with economic and cultural differences is organizational structures between developed and developing nations. Therefore, we believe that the impact of HRM on CSR and performance would be stronger for developed countries as compared to developing countries due to strong organizational structure.

### Population

A highly structured questionnaire was used to collect the respondents working in 3-star, 4-star, and 5-star hotels in the UK, Italy, and Pakistan. The first step is to extract the number of hotels in each star category from all countries. For instance, 12,089 hotels list was extracted from 52 Chamber of Commerce situated across the UK. The total number of hotels in Italy is 33,000, while 24,200 hotels fall in three-, four-, and five-star categorization. Lastly, the list of 475 hotels in Pakistan was prepared from different sources such as the Hotel Association of Pakistan, Government Data, and indexing website.

### Sampling strategy and sampling size

The lists of hotels from each country were prepared and developed separate strata for each country. Using the basic criteria of identifying sample size, 10% of hotels from the UK (1208) and Italy (2420) were randomly selected using a 1% margin of error and a 95% confidence interval strategy. All hotels were chosen from Pakistan as it contains fewer hotels as compared to their counterparts. To infer sample size from the literature, Tinsley and Tinsley (1987) also proposed the minimum sample size to have 5 to 10 respondents for each item, while Hair et al. (1998) suggested that the sample should be in between 5 and 20 respondents against each item. We measured HRM, CSR, and sustainable performance using 30 items; therefore, the adequate sample is in between 150 and 600.

We send the questionnaire and cover letter to 1,208 UK hotels, 2,420 Italian hotels, and 475 Pakistani hotels. Considering the study’s objectives, a single respondent strategy was adopted to get precise information about HRM, CSR, and sustainable performance. After multiple follow-ups and deleting questionnaires with missing values and outliers (e.g., opting strongly agree with all questions), we successfully collected the data of 438 UK, 520 Italians, and 354 Pakistani hotels. A total of 1,312 questionnaires were received and exceeded the upper limit of minimum sample size.

### Sample representativeness

We used the Chi-square test to determine the sample representativeness to compare the sample to population ratio, as no official statistics about the number of employees working in the selected hotels. The Chi-square tests were insignificant (χ2 = 2.389, *p* = 0.342; χ2 = 1.017, *p* = 0.135), hence indicating that the sample is representative to the population.

### Non-response bias

Non-response bias between early and late respondent was determined using Armstrong and Overton’s (1977) proposed method. The results showed no differences in age (χ2 = 0.04, *p* > 0.10), experience in current organization (χ2 = 1.36, *p* > 0.10), designation (χ2 = 2.01, *p* > 0.10), and experience in hospitality industry (χ2 = 0.09, *p* > 0.10). These results indicate no issue of non-response bias.

### Common method bias

We examined the relationship of HRM and CSR with sustainable performance in the hotel industry of culturally and economically different countries since we use a self-reported measure which may lead to a common method bias (CMB) issue. As Podsakoff et al. (2003) proposed, Harman’s one-factor test was run to address procedural and statistical remedies to address CMB. The results revealed that the one-factor model explains 39.45% variance (less than 50%), indicating that CMB is not an issue in this research.

### Data analysis

The data were analyzed using SPSS 22.0 and AMOS 21.0. We performed confirmatory factor analyses and Cronbach’s alpha to determine discriminant validity, convergence validity, and reliability. Additionally, correlation and path analyses were employed for hypotheses testing, while multigroup analyses (MGA) were performed to identify the differences in three-, four-, and five-star hotels.

### Measuring scale

The scales used to measure HRM, CSR, and sustainable performance were extracted from previous studies and adapted for this study. Before administering the questionnaires at a large scale, a pilot study was conducted to determine the reliability and validity of the instruments, and results show acceptable values for reliability (< 0.70) and validity (< 0.60). HRM was measured on a 14-items scale, including questions related to cross-functional teams, remuneration systems, training, employee rights, safety procedures, well-being, recruitment, rules, and regulations extracted from previous studies (Kim et al., 2019; Shen & Benson, 2016). The exploratory factor analysis (EFA) was performed on the HRM variable. The result indicates one factor that explains 56.6% variance. In next step, the confirmatory factor analysis (CFA) was performed that also shows unidimensional construct with acceptable fit indices (χ^2^/df = 344.88/117, *p* = 0.001; SRMR = 0.04, RMSEA = 0.05; NFI = 0.95, IFI = 0.94, CFI = 0.95). Moreover, the factor loading of each item was greater than 0.60, and the composite reliability was 0.86.

The CSR-related initiatives of the hospitality industry are measured on a 9-items scale that includes questions on economic, environmental, and social development adopted from the previous study (Turker, 2009). EFA was performed on the CSR variable, and the result shows a unidimensional variable that explains 54.3% variance. In subsequent CFA analysis, the result endorses the unidimensional variable with acceptable fit indices (χ^2^/df = 310.53/105, *p* = 0.001; SRMR = 0.05, RMSEA = 0.06; NFI = 0.93, IFI = 0.95, CFI = 0.95). Additionally, the factor loading of each item was greater than 0.60, and the composite reliability of CSR was 0.84.

Lastly, sustainable performance was measured on a 7-items scale that includes questions on economic performance, social performance, and environmental-related performance (Zhu & Sarkis, 2004; Bai & Sarkis, 2010; Nikolaou et al., 2013). EFA analysis on sustainable performance confirms unidimensional construct that explains 53.9% variance, whereas the CFA also endorses the EFA results with acceptable fit indices (χ^2^/df = 347.06/126, *p* = 0.001; SRMR = 0.05, RMSEA = 0.05; NFI = 0.94, IFI = 0.94, CFI = 0.96). All items were measured on a seven-point Likert scale where “1 = strongly disagree” and “7 = strongly agree.” The questionnaire also includes the demographic profile of the respondents, such as gender, position, and experience in the industry, and hotel characteristics like the number of rooms, hotel stars, and years of operation. Moreover, all the scales are given in Appendix A.

## Findings

### Demographic profile and hotel characteristics

The demographic profile of the respondents and hotels’ characteristics is presented in detail in Table 1. Most of the hotels (55%) have a 3-star category, followed by 4-stars (38%) and five-stars (7%) hotels. Regarding hotel size, 51% of hotels have rooms ranging from 21 to 50, whereas 37% exist between 11 and 20 years. Concerning gender, 65 were male, and 50% of respondents had ages between 36 and 45 years of age. 56% of respondents were working on managerial rank, 21% were Chief Executive Officers, and 4% of respondents owned the hotels. For experience results, 41% of respondents worked in the same position from 4–6 years, while 42% worked between 7 and 9 years on the current designation. Moreover, 48% of respondents had 7–9 years in the current hotel where they are working, while 35% had an experience of 4–6 years in the said hotel.

**Table 1:** Demography and hotel characteristics

Variable		Frequency	Percentage
*Demographic statistics*			
Gender	Female	456	34.8
	Male	856	65.2
Age (in years)	18–25	22	1.7
	26–35	308	23.5
	36–45	656	50.0
	46–60	288	22.0
	61 +	38	2.9
Designations	Chief Executive Officer	270	20.6
	First Level Employee	126	9.6
	Manager	728	55.5
	Middle Manager	128	9.8
	Owner	56	4.3
	Other	4	.3
Years of experience in current position (in years)	1–3	106	8.1
	4–6	534	40.7
	7–9	552	42.1
	10–20	82	6.3
	20 +	38	2.9
Years of Experience in Current Hotel (in years)	1–3	82	6.3
	4–6	452	34.5
	7–9	630	48.0
	10–20	110	8.4
	21 +	38	2.9
Years of experience in hospitality industry (in years)	1–3	38	2.9
	4–6	138	10.5
	7–9	518	39.5
	10–20	386	29.4
	21 +	232	17.7
Hotel characteristics			
Country respondents	Italy	520	39.6
	Pakistan	354	27.0
	UK	438	33.4
Hotel star	3-Star/ 3-Star Superior	724	55.2
	4-Star/ 4-Star Superior	494	37.7
	5-Star/ 5-Star Superior	94	7.2
Hotel size	2–20 Rooms	82	6.3
	21–50 Rooms	676	51.5
	51–100 Rooms	482	36.7
	101 + Rooms	72	5.5
No. of Employees	0–9	42	3.2
	10–49	680	51.8
	50–249	522	39.8
	250 +	68	5.2
Year of Establishment	1–5	54	4.1
	6–10	312	23.8
	11–20	486	37.0
	21 +	460	35.1

### Measurement model

Before testing the hypothetical model, a series of CFAs were performed using AMOS 20 to determine the model fitness in overall data and individual for each hotel category. As it indicates, three-factor model of HRM, CSR, and sustainable performance show adequate model fitness (χ2/df = 1658.617/557; *p* < 0.001, SRMR = 0.06; RMSEA = 0.05; NFI = 0.94; IFI = 0.94; CFI = 0.95) as compared to alternative models. Similarly, the measurement model for each hotel categorization shows adequate fitness. The reliabilities (Cronbach’s alpha and composite reliabilities) and validities (average variance extraction, inter-factor correlations, and factor loading) show that all the values are greater than the minimum threshold as Hair et al. (2012) propose. The summary of these statistics is given in Table 2.

**Table 2:** Measurement indices, reliabilities and validities

		HRM	CSR	Sustainable performance
FL Range	3-stars	.75 – .84	.73 – .81	.76 – .83
	4-stars	.74—.80	.72—.77	.79—.84
	5-stars	.81 – .84	.76—.82	.72—.80
	Overall	.76—.82	.73—.80	.75—.82
α	3-stars	0.84	0.79	0.78
	4-stars	0.80	0.81	0.83
	5-stars	0.82	0.80	0.85
	Overall	0.82	0.8	0.82
CR	3-stars	0.85	0.80	0.79
	4-stars	0.83	0.82	0.83
	5-stars	0.85	0.82	0.84
	Overall	0.84	0.81	0.82
AVE	3-stars	0.64	0.69	0.70
	4-stars	0.68	0.70	0.71
	5-stars	0.71	0.68	0.69
	Overall	0.68	0.69	0.70
Fit indices	3-stars	χ2/df = 1537.49/515; *p* < 0.001, SRMR = 0.05; RMSEA = 0.05; NFI = 0.96; IFI = 0.94; CFI = 0.96		
	4-stars	χ2/df = 1654.22/553; *p* < 0.001, SRMR = 0.06; RMSEA = 0.06; NFI = 0.95; IFI = 0.95; CFI = 0.95		
	5-stars	χ2/df = 1784.11/599; *p* < 0.001, SRMR = 0.06; RMSEA = 0.07; NFI = 0.95; IFI = 0.94; CFI = 0.95		
	Overall	χ2/df = 1658.617/557; *p* < 0.001, SRMR = 0.06; RMSEA = 0.05; NFI = 0.94; IFI = 0.94; CFI = 0.95		

*FL* = *factor loading, a* = *Cronbach’s alpha, CR* = *composite reliability, AVE* = *average variance extraction*

### Descriptive and correlations

Table 3 shows the descriptive statistics and correlations among HRM, CSR, and sustainable performance for overall and hotels’ category-wise. As table indicates, the correlation between HRM with CSR (*r* = 0.43, *p* = 0.001), HRM with sustainable performance (*r* = 0.34, *p* = 0.001), and CSR with sustainable performance (*r* = 0.72, *p* = 0.001) is higher for hotels with 5-star category followed by 3-stars and then 4-stars.

**Table 3:** Descriptive and correlations

	M	SD	Overall			3-Stars			4-Stars			5-Stars		
			*1*	*2*	*3*	*1*	*2*	*3*	*1*	*2*	*3*	*1*	*2*	*3*
1. S.P	5.01	1.01	1.00			1.00			1.00			1.00		
2. CSR	5.27	0.94	.64	1.00		.64	1.00		.63	1.00		.72	1.00	
3. HRM	5.49	0.97	.22	.34	1.00	.23	.34	1.00	.18	.32	1.000	.34	.43	1.00

*M* = *mean, SD* = *standard deviation, S.P.* = *sustainable performance. All correlations values are significant at 0.001 level*

### Hypotheses testing

**Fig. 2:**
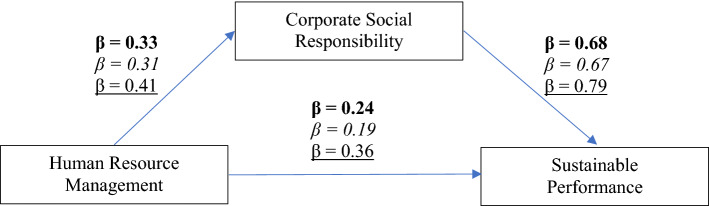
Multigroup analysis

The hypotheses for this study were tested using structural equation modeling (SEM), and a summary of the results is shown in Table 4 and Fig. 2. For H1 and H2, the results indicate that HRM has positive influence on CSR (beta = 0.33, *p* = 0.001) and sustainable performance (beta = 0.23, *p* = 0.001). The results also confirm that CSR has positive relationship with sustainable performance (beta = 0.69, *p* = 0.001). Therefore, the results confirm the H1, H2, and H3 in the overall data. Additionally, the mediating role of CSR in HRM and sustainable performance is also significant (*p* = 0.05), hence supporting H4.

**Table 4:** Summary of SEM

Hypothesis	overall					
	*Coefficient*			*SE*		
H1: HRM CSR	0.33***			0.025		
H2: HRM S.P	0.23***			0.028		
H3: CSR S.P	0.69***			0.022		
H4. HRM CSR S.P	0.19**			0.026		
	3-Stars		4-Stars		5-Stars	
	*Coefficient*	*SE*	*Coefficient*	*SE*	*Coefficient*	*SE*
H5: HRM CSR	0.33***	0.034	0.31***	0.041	0.41***	0.090
H6: HRM S.P	0.24***	0.037	0.19***	0.045	0.36***	0.091
H7: CSR S.P	0.68***	0.030	0.67***	0.037	0.79***	0.082

^***^ Sig at 0.001 level; ** Sig at 0.05 level

Table 4 also indicates the results for each hotel’s category to examine the relative differences between the impact of HRM on CSR and sustainable performance. As mentioned in the table, the impact of HRM on CSR (beta = 0.41, *p* = 0.001) and sustainable performance (beta = 0.36, *p* = 0.001) is relative stronger for the hotels with 5-stars ranking. Surprisingly, three-star hotels have relatively stronger results for each relationship. Hence, the results also supported H5, H6, and H7.

We run separate analyses to examine the cultural differences between the relationship of HRM, CSR, and sustainable performance. As shown in Table 5, significant cultural differences were found between the relationships of HRM, CSR, and sustainable performance in hospitality industries of Italy, UK, and Pakistan. For instance, the impact of HRM on CSR is relatively stronger (beta = 0.670, *p* = 0.001) and HRM impact on sustainable performance (beta = 0.435, *p* = 0.001) in UK sample as compared to other countries. Additionally, CSR is more strongly related to sustainable performance in Pakistani sample (beta = 0.753, *p* = 0.001).

**Table 5:** Cultural differences

Hypothesis	Italy *β*	UK *β*	Pakistan *β*
HRM CSR	0.510***	0.670***	0.380***
HRM S.P	0.372***	0.435***	0.396***
CSR S.P	0.599***	0.661***	0.753***

^***^ Sig at 0.001 level

## Discussion and conclusion

The goal of the present study was to improve our understanding of the role of HRM and CSR in maintaining the sustainable performance. We theoretically account for the implications of HRM and CSR strategies and activities on the generation of sustainable performance. We believe that HRM facilitates the employees in designing and executing of sustainable practices and that subsequently leads to higher economic, environmental and social performance, as supported by previous studies (Kim et al., 2019; Shafaei et al., 2020). Regarding CSR influences, the direct and significant relationship with sustainable performance also endorsed the previous studies (Franzoni et al., 2021). Considering the results, it is evident that the firms are learning new techniques to work in the current pandemic of COVID-19 and achieve their goals effectively and efficiently. Apart from the pandemic, the organizations face different issues such as environmental constraints, legal systems, socio-economic problems, and resources. Many industries, particularly hospitality and tourism, face a huge backlash from various stakeholders to address sustainability issues in today's environment. Hence, it is time for hoteliers to adopt sustainability-related initiatives to reduce their influence on water consumption, clean energy, global warming, poverty reduction, and responsible production (Pham et al., 2020; Úbeda-García et al. 2021).

### Theoretical implications

The results supported the RBV theory that claims that HRM and CSR are resources that not only increase sustainable performance but also act as a source of competitive advantage. This study from the hospitality industry on HRM, CSR, and sustainable performance proposes imperative theory implications. First, based on RBV theory, the findings confirm the role of HRM and CSR in achieving sustainable performance. Five-star hotels with competing resources and capabilities strengthen them to engage in CSR initiatives by proposing HRM policies.

The results reported that HRM has a stronger influence on CSR and sustainable performance for the hotels with a 5-star ranking, but interestingly, 3-star hotels compete more strongly than 4-star hotels. We believe that the 3-star hotels are working more strongly to create a sustained competitive advantage by promoting CSR-related policies and effective implementations through HRM. We are also convinced that HRM and CSR support's strategic role significantly contributes to the hotel’s competitiveness, especially for 3-star hotels. From the RBV perspective, we consider HRM or human capital, and CSR leads to sustainable competitive advantage. Hence, these results provide vital support to the debate linked with CSR and HRM with organizational success (Barney & Wright, 1988). Under RBV, both HRM and CSR are perceived as strategic assets that contribute to long-term profitability and competitiveness and build a strong reputation among consumers and employees’ minds. Hence, hoteliers need to pursue organizational schemes that replenish, support, and exploit HRM and CSR resources to achieve a competitive advantage over competitors.

Despite enormous advantages to unearth HRM–CSR–performance nexus, few studies have empirically examined this relationship (Jamali et al., 2015; Shafaei et al., 2020) and few in the hospitality industry (Kim et al. al. 2019; Umrani et al., 2020). But no study has been conducted to see how the hotels’ categorization played its role in developing HR policies that effectively implemented CSR initiatives and helped achieve sustainable performance. Park and Levy (2014, p. 36) suggest that “the lack of managerial awareness and learning in the CSR arena has been argued to be a major organizational barrier to implementing socially responsible practices.” Therefore, this study fills a significant gap in understanding the differences in HRM–CSR–sustainable performance relationships within the hospitality industry. It will help the researchers understand the basic assumptions of intended practices developed within an organizational context by considering their resources and capabilities.

### Practical implications

This study also offers a few worthy managerial implications. First, the relative stronger influence of HRM on CSR and sustainable performance for five-star hotels reveals that HRM practices of such hotels motivate its employees to show positive intentions toward CSR initiatives and their efforts in achieving sustainable performance. In this situation, it is recommended that the managers from other hotels’ categories, especially four-star hotels, develop widely appreciated HRM policies and effective implementation to achieve CSR and performance-related initiatives. The managers can develop knowledgeable and skilled staff to deliver quality services and meet customer expectations for higher sales growth and customer satisfaction through these policies. Secondly, the hospitality industry should consider HRM as a strategic partner, irrespective of hotels’ stars, as HRM practices played their role in effectively executing CSR plans and increasing financial, social, and environmental performance. Lastly, managers should arrange knowledge-intensive training programs about CSR and sustainability to create awareness among employees and their roles in environmental protection.

### Limitations and avenue for future studies

This study has some significant limitations that will help scholars to overcome in their future studies. First, the data were collected from one respondent in each selected hotel, and secondly, subjective measures were used to collect the data on HRM, CSR, and sustainable performance. These limitations have concerns related to common method bias and single informant. Therefore, future researchers should collect the data from multiple sources from each hotel to avoid common method bias and obtain different perspectives. Third, the data were collected from one industry of three countries. It is recommended that future researchers should conduct this research in other sectors and report cross-cultural differences (if any) as well. Additionally, the data were cross-sectional; therefore, longitudinal studies should be conducted to establish causal relationships. Lastly, future researchers should use control variables such as hotel star ranking, year of operations, and hotel size in the theoretical model.

### Conclusion

This research is an important study to examine the impact of HRM on CSR and sustainable performance and CSR’s influence on sustainable performance in the hospitality industry of the UK, Italy, and Pakistan. The data were analyzed using SEM on the overall sample and separately for each hotel category, i.e., three-star, four-star, and five-star hotels. Overall, the results supported all the hypotheses and confirmed that the impact of HRM on CSR and sustainable performance and CSR’s influence on sustainable performance is different in each hotel category. The association of HRM with CSR and sustainable performance is relatively stronger in five-star hotels, followed by three-star and four-star hotels. Theoritically, this study extends the existing literature on the relationship between HRM, CSR, and performance (Umrani et al., 2020; Úbeda-García et al. 2021). In terms of practical implications, we propose that four-star and five-star hotel should reconsider their approach toward CSR and sustainability initiatives as three-star hotels are performing relatively more positively.

## Data Availability

The datasets generated during and/or analyzed during the current study are available from the corresponding author on reasonable request.

## Appendix A

### Corporate social responsibility

1. The hotel works for social, environmental, and economic development rather than focusing on profit maximization only
2. CSR policies need to be considered as a core and inseparable component of the overall service or product offering
3. Implementing CSR in business means that the managers must comply with the rules
4. The hotel has CSR policies to head off potentially disastrous consumer backlashes
5. The hotel selects suppliers or business partners based on CSR criteria
6. The hotel selects suppliers or business partners based on financial criteria
7. Charitable contributions are fundamental to the concept of CSR. The hotel must make charitable contributions every year
8. The hotel encourages partnerships with local businesses (local suppliers) and local organizations (schools, universities, local public authorities, etc.)
9. The hotel promotes local heritage (cultural, historical, arts and crafts, etc. …)

### Human resource management

1. The hotel has a code of conduct related to CSR policies that every employee must know
2. The hotel carefully hires employees who show a positive mindset toward CSR practices.
3. The remuneration system is based on the achievement of economic performance
4. The remuneration system is based on the achievement of sustainable (economic, social, and environmental) performance
5. The hotel ensures the balanced representation of women within their decision-making bodies and memberships
6. The principle of equal remuneration for men and women workers for work of equal value must apply
7. The cross-functional teams or steering committees (i.e., CSR committees) are essential for better coordination related to CSR practices
8. The proper training and development of employees leads to better CSR implementations
9. Managers make the majority of decisions without consulting employees
10. Managers share the strategic plan with employees
11. Managers share the economic and social-environmental results with all stakeholders
12. Solving complex problems requires a complicated, male approach
13. The hotel ensures all the safety procedures for employees
14. The hotel ensures the implementation of policies to respect human rights

### Sustainable performance

1. The hotel could launch, maintain, and develop a prevention strategy as a key factor of a policy for sustainable development to protect the environment and its deterioration
2. The hotel management is committed to reducing environmental fines
3. The Board recognize CSR as fitting within their corporate governance role and responsibility for social, economic, and environmental performance
4. Have a section on CSR under its annual report to indicate the social, economic, and environmental performance
5. Environmental policies and practices are significantly contributed to sales growth
6. The levels of tourists’ satisfaction contributed to sales growth
7. The levels of employees’ satisfaction contributed to sales growth
